# Doctoris Medici Nemausensis, Dissertatio Epistolaris de Cicutâ, Strammonio, Hyoscyamo, et Aconito

**Published:** 1781-11

**Authors:** 


II. Johannis Razoux, *Doctoris Medici Nemausensis, Dissertatio Epistolaris de Cicutâ, Strammonio, Hyoscyamo, et Aconito.* 8vo. Nismes, 1780. 46 pages.

THIS dissertation is addressed to Baron Storck. In the first section the author relates several cases in proof of the efficacy of hemlock. Of these cases some few were communicated to him by his friend Dr. Pons, physician at St. Gilles in Languedoc: the rest are from his own practice. The following seem to be the most interesting:

A woman, aged forty years, had for a long time been subject to vomitings, which returned so frequently that at length she was seldom free from them. The efforts to vomit became more and more violent; she complained of a painful oppression at her stomach, and her nights were sleepless. After trying a variety of remedies without any good effect, our author prescribed the extract of hemlock. The patient began with small doses of this medicine, which she increased by degrees. In two months the vomitings ceased, and her digestion and health were soon perfectly restored.

A young ecclesiastic, by devoting himself too closely to study, lost his appetite, and became lanlanguid and melancholy. After taking a purgative medicine, he was adviſed to ſwallow hemlock pills every morning, and to waſh them down with a tea-cupful of an infuſion of the ſame plant. At the end of ten or twelve days he began to experience a ſenſation of heat at his ſtomach, and it was not long before he was perfectly recovered.

A young woman, in her twenty-fifth year, was attacked with a fever, which terminated in aſcites. Her pulſe was ſmall and quick, but ſomewhat tenſe. She voided only a ſmall quantity of a turbid, high-coloured urine. Her appetite was almoſt entirely gone, her countenance and ſtrength ſunk, and ſhe complained of a conſtant thirſt. Diuretics and purgatives having been employed in a variety of forms without affording her any apparent relief, the author had recourſe to the extract of hemlock, interpoſing a purge every fifth or ſixth day. After purſuing this courſe about two months, the patient began to diſcharge a great quantity of urine, her belly leſſened, and in a ſhort time ſhe found herſelf well.

Dr. Razoux likewiſe makes mention of a caſe of jaundice, accompanied with an enlargement of the ſpleen, in a woman thirty years old, whoſe menſes had been ſuppreſſed three years; and of a man afflicted with rheumatiſm, and ſeveral fiſtulous lous ulcers: both theſe patients were cured by hemlock.

In the ſecond ſection the author treats of the effects of ſtrammonium. He relates only a ſingle caſe in which a cure was effected by this medicine. The patient was a young man, aged twenty-two years, who had been four years ſubject to epilepſy. The fits at the beginning returned only once a month; but afterwards once, twice, and even thrice a week. The convulſions were irregular and frequent. In other reſpects the patient was well; his pulſe and appetite were good, and his ſleep tranquil. Several remedies had been tried without ſucceſs. The author began by preſcribing half a grain of the extract of ſtrammonium twice a day, and gradually increaſed the doſe till the patient took two grains a day. The fits ſoon became leſs frequent, and at length were entirely removed. Dr. Razoux acknowledges, that in ſeveral other caſes in which he tried this remedy, it did not prove equally ſucceſsful, which he ſuppoſes to have been in ſome meaſure owing to the irregularity of the patients, or the obſtinacy of the diſeaſe.

The external uſe of the leaves of hyoſcyamus has long been known, but this plant was ſeldom adminiſtered internally before Storck ventured to preprefcribe it.—Dr. Razoux afferts, that the extract of henbane is grateful to the nervous fyftem, allays pain, ftrengthens the ftomach, induces fleep, and is a powerful antifpafmodic. One of his patients, a man forty years of age, made ufe of the frefh leaves of henbane as a topical application to a painful fwelling. A maid fervant in preparing fome broth for him, by miftake mixed fome of thefe leaves with other herbs. The patient was immediately feized with a fenfe of coldnefs. He was unable to ftir, and his pulfe could hardly be felt. He complained of a painful fenfation of heat in his throat and fauces, and his vifion was extremely confufed. A weaknefs of fight lafted even a month after his recovery. He pronounced his words with difficulty, and his memory feemed to have fuffered; but he did not lofe his underftanding; neither was he convulfed. An emetic, diluting liquors, and purgative medicines, foon removed thefe alarming fymptoms; after which, by the advice of our author, the patient took fmall dofes of the extract of henbane, which compleated the cure; fo that this medicine, as he obferves, employed with prudence, ferved to remove the very evils it had occafioned. This is the only inftance he gives of its good effects.

We fhall content ourfelves with noticing the laſt of three caſes related in the fourth ſection, to prove the efficacy of the *extract. aconit.* prepared with ſugar, two grains of which contain half a grain of the *aconitum.* The patient, who was a woman fifty years of age, after having been for ſeveral months ſubject to rheumatiſm, was attacked with an acute fever, which yielded to venæſection, purges, and other remedies; but ſoon afterwards the pains returned with increaſed violence. At this period of the diſeaſe our author began with giving her two grains of the extract once a day. In ten days the pains were diminiſhed, but the patient had had no evacuation by ſweat or otherwiſe. On the twelfth her belly became tenſe and painful. The uſe of the extract was ſuſpended, till theſe ſymptoms were removed by proper evacuations, when ſhe returned again to the aconitum, which in ſeven or eight days brought on the ſame bowel complaints as before, but they ſoon yielded to the former remedies. After this ſhe was able to continue the uſe of the extract for a month without any inconvenience, and at the end of that time the rheumatic ſymptoms were entirely removed.

The fifth and laſt ſection relates to the *colchicum.* The author informs us that his experiments with this medicine are not yet ſufficiently numerous to

 enable enable him to ſpeak deciſively of its efficacy. Of three dropſical patients to whom he adminiſtered it, only one obtained a cure by it; but its effects in the other two caſes proved it to be a powerful diuretic.

